# Augmented reality navigation systems in endoscopy

**DOI:** 10.3389/fgstr.2024.1345466

**Published:** 2024-05-22

**Authors:** Rebecca Metzger, Per Suppa, Zhen Li, Anant Vemuri

**Affiliations:** ^1^ Digital Unit, Olympus Europa SE & CO. KG, Hamburg, Germany; ^2^ Technology Innovation, Olympus Surgical Technologies Europe, Hamburg, Germany; ^3^ Advanced Technology Research, Olympus Corporation, Tokyo, Japan

**Keywords:** artificial intelligence, endoscopy, laparoscopy, gastroenterology, digital health, augmented reality, computer aided detection (CADe)

## Abstract

Navigation assistance has become part of our daily lives and its implementation in medicine has been going on for the last 3 decades. Navigation is defined as the determination of a position in space in the context of its surroundings. While routing applications used in cars highlight the street to follow, in medical applications the real-world perception of the user is enriched by digital elements that provide guidance and help the user navigate. Unlike Virtual Reality (VR) solutions, which are mainly used for training, Augmented Reality systems (AR) do not require the user to wear specific headsets/goggles, but the virtual elements are overlaid over the real image displayed on a monitor. Depending on the application and the indication, there are large differences in how much these solutions have entered clinical routine. Especially in the fields of GI endoscopy and laparoscopy, AR navigation solutions are less prominently employed in clinical practice today and still hold significant potential to elevate patient care by improved physician support. This review touches upon why AR navigation systems are currently just starting to become implemented in the GI/laparoscopic clinical routine and which applications can be expected in the GI endoscopy and laparoscopy field. By taking the practitioner’s perspective and following the intuitive navigation workflow, it gives an overview of major available and potential future AR-applications in the GI endoscopy and laparoscopy space, the respective underlying technologies, their maturity level and their potential to change clinical practice.

## 1 Introduction

Since the invention of the first endoscopes, several innovations have revolutionized the examination of the interior of the gastrointestinal tract in order to diagnose and treat disease. High-performing lens systems, flexible fiberoptics and digital video endoscopy systems have enabled endoscopy to become a standard procedure for interventional diagnosis and treatment of various disease states and abnormalities that otherwise would remain undetected and untreated (1, 2).

Supporting detection and optimal treatment is the main goal of computer aided technologies that have been developed in the last decade with first products having reached market maturity in the last years. These tools have in common that they employ Augmented Reality (AR) by combining digital elements, generated by computer graphic systems with the real-world view (3). Often the additional information is superimposed onto the real-world image on the same screen, thereby allowing the practitioner to gain a deeper understanding of the respective environment and to build advanced intuition (4). The algorithms used for the generation of the digital elements, nowadays mostly employ artificial intelligence, sometimes referred to as AI-XR solutions (5).

Benefits of AR navigation systems range from superior workflows with shorter procedure times to improved patient outcomes e.g., due to minimized safety margins when excising tumorous tissue (6–9).

Most technical process and implementation in the clinics can be observed in disciplines like neurosurgery, orthopedics or dentistry, less in the GI/laparoscopy field (10, 11). In laparoscopy or GI endoscopy, AR navigation applications are still limited to less complex tasks, in comparison to solutions that are part of e.g., the neurosurgery routine today (10).

While the question “where am I” and the need to recognize objects or structures is a commonality across procedures, there are major differences in feasibility and user-value of the respective AR application. In GI endoscopy the position of the object of interest, such as a polyp, is often unknown and clearly visible landmarks are rare (e.g., in the colon). In laparoscopic surgery, the target and its position is known, e.g., a tumor that has been pre-operatively identified by other imaging means. On the other hand, the best path to the target is unknown in laparoscopy, while it is directly given in GI endoscopy by the gut lumen.

Thus, the respective value-add and challenges of a certain AR navigation solution highly depend on the procedure and related pain points.

A common challenge for all AR solutions is the risk of distracting the user from critical aspects of procedure. Situational information needs to be inserted into the procedural workflow in a way that prevents loss of focus or mental fatigue of the user. Current regulations require risk mitigation strategies that can include user-centric design with on-demand, prioritized information display, thorough user education and an intuitive, integrated control of the AR system.

As soon as image information is analyzed as input for an AR application, common challenges include imaging artifacts such as blurriness, low contrast, suboptimal exposure with reflections and occlusions or procedure-related confounding factors, such as smoke from energy devices or bleeding. Advanced imaging technologies, such as narrow band endoscopic imaging (NBI), Texture and Color Enhancement Imaging (TXI) as well as hardware solutions that aim at reducing occlusions (e.g., assisted colonoscopy devices like Micro-Tech Embrella or Olympus EndoCuff Vision**™**) and molecular imaging technologies lead to a more informative real-time image that allows for better analyses and higher quality results.

Complexity increases when multi-modal imaging data needs to be overlaid to enrich the real-time image with information that is otherwise invisible to the user. When complementary data is generated during the procedure, generating overlays is generally easier as the organ shape is identical for the different imaging modalities (e.g., combining intra-operative CT with a real-time laparoscopic image). However, this data is often difficult to obtain during GI endoscopy/laparoscopic procedures as typical procedure rooms lack the respective equipment in clinical practices today, unless they are used for very specific GI endotherapy procedures, such as Endoscopic Retrograde Cholangiopancreatography (ERCP). The combination of pre-operative imaging data with the real-time view during the procedure can be highly demanding for soft tissue, as the organ’s shape significantly changes between the imaging timepoints.

The challenge of organ deformation is also the key reason why AR navigation solutions are much further advanced in disciplines that focus on rigid organs, such as orthopedics, neurosurgery and dentistry compared to laparoscopy and GI endoscopy used to examine and treat pliable organs. Heartbeat, respiration, laparoscopic insufflation pressure and physical probing lead organs like the intestine to change in their shape during procedures. This drastically complicates image reconstruction (creating a visual representation) and object recognition efforts. Other disturbance factors, such as fast movements or the use of a water-jet during endoscopy add to this.

However, technical advancements, especially progress in the AI model space, heavily support dealing with these complexities and pave the way for AR navigation solutions becoming part of tomorrow’s clinical routine.

Despite the challenges in adopting AR to the GI/laparoscopic field, this review aims to describe a path towards a stepwise implementation of AR applications into clinical routine with the motivation to render procedures more efficient and increase patient outcome.

### 1.1 Cognitive navigation loop and AR level concept

AR navigation support enriches the real-time image with experience-based, situational and contextual information that helps the user to navigate. AR solutions can support the user at different steps of the cognitive process of a navigating endoscopist or surgeon.

Whenever the situation in the field of view is changing, the following cognitive loop (simplified) is passed through see **Table 1**.

**Table 1:** Cognitive navigation loop.

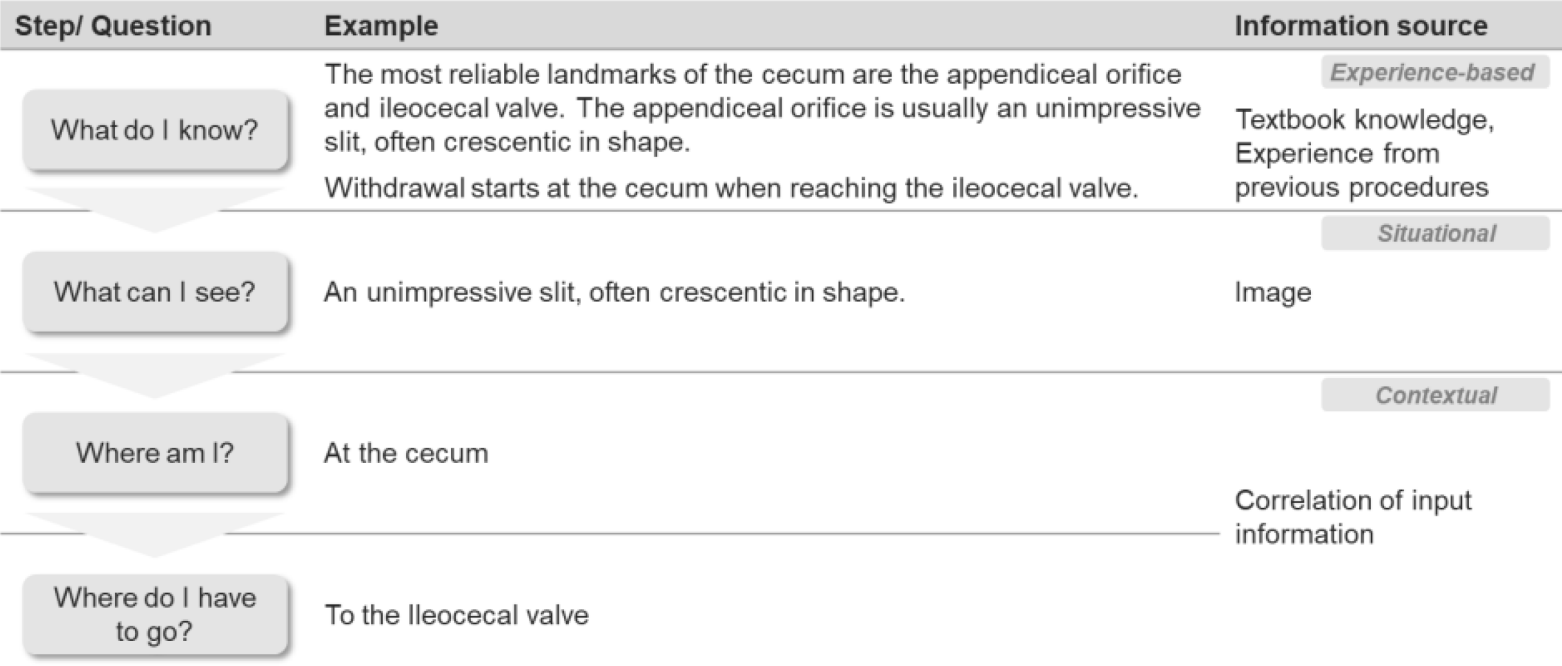

The questions can be answered by the practitioner by gathering of “experienced-based”, “situational” and “contextual” information, whereby all information sources build on each other. An experience-based information is required to understand situational information and the latter is required to understand contextual information. AR navigation systems can be categorized into 3 levels along these lines.

## 2 AR applications in GI endoscopy and laparoscopy

### 2.1 Applications of level 0 AR/”What do I know?”

Provision of information that is independent from the real-time endoscopic video image and needs to be correlated to the real-time image by the user (see example in **Table 2**).

**Table 2:** AR application levels.

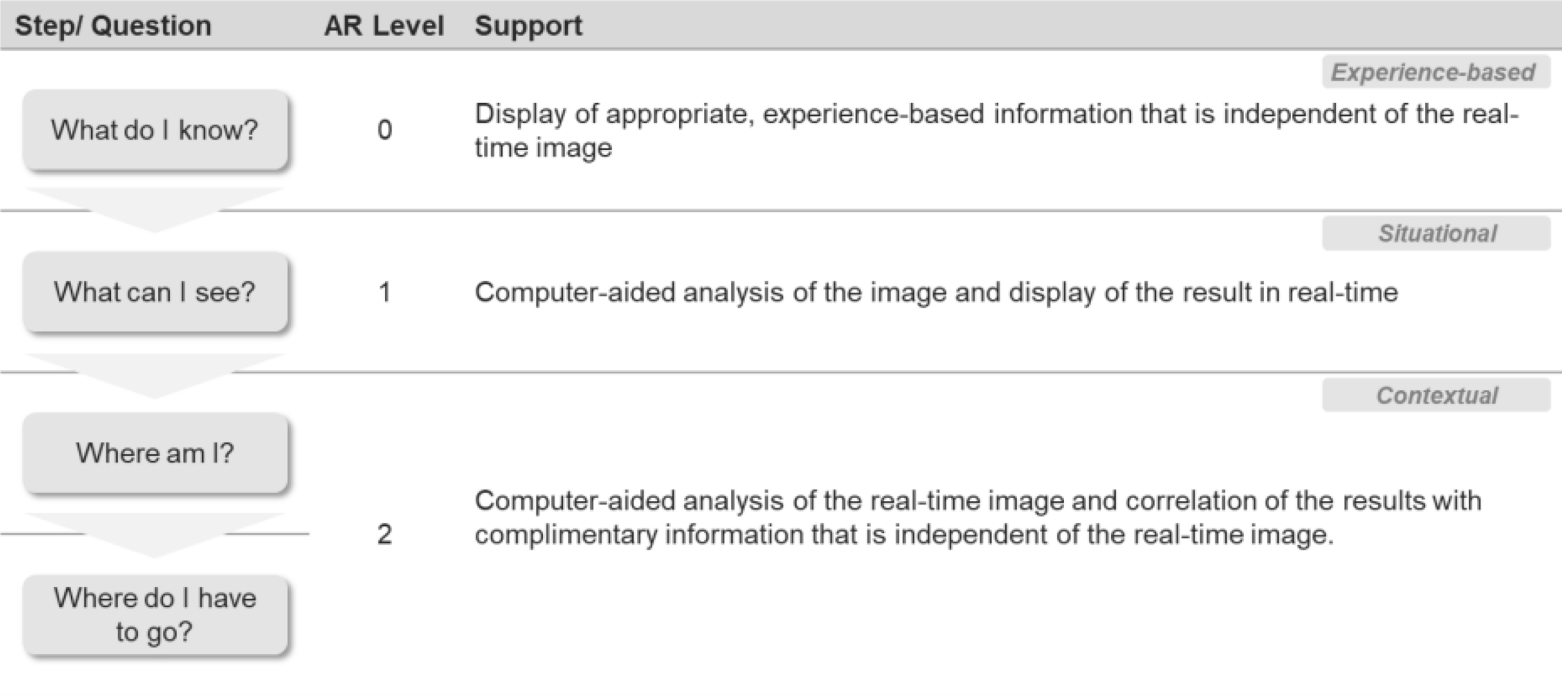

#### 2.1.1 Procedure instructions

Information that complements the endoscopic image is displayed on the endoscopy monitor to aid the user in performing the procedure. Steps of a procedure and e.g., related anatomical landmarks that are displayed.

Structured text databases are used to provide standardized procedure instructions and safety check-lists. These applications are found on the market today (e.g., within iOR systems), as well as more simple solutions, such as withdrawal timers for colonoscopy. As long as the procedures are largely standardized, the number of supported procedures can be easily extended by adding the respective textbook-knowledge to the underlying databases.

#### 2.1.2 Patient information

Patient information that complements the endoscopic image is displayed on the endoscopy monitor to highlight specific characteristics (such as aberrant anatomy) that help the practitioner to navigate.

Interfaces with the patient record management system are needed for displaying patient-specific information from previous examinations during the procedure. To date, the integration level with the EHR systems is limiting the depth of available information to mostly only basic patient biodata. Further developing available clinical text mining algorithms can allow for only displaying the pieces of information that are most relevant for the procedure (12). The technical aspects of data integration and further analyses are less limiting than the regulatory situation, as EHR patient data is highly sensitive and data security and privacy are concerns that need to be addressed in an environment of increasing requirements (13).

#### 2.1.3 Static 3D organ model

A pre- or intraoperatively acquired patient-specific organ model is displayed on the monitor to support the practitioner in correlating pre-operative imaging data with the real-time endoscopic image. The organ model can be oriented manually, but does not sync with the real-time organ orientation.

Pre-procedure imaging information, such as CT image data is used to generate a 3D organ model. CT imaging is rarely combined with GI endoscopy and the value of a 3D organ model is limited for cavity examinations (such as colon, stomach, bladder). For laparoscopic procedures, however, value and market maturity are high. Several solutions employing static 3D models assisting in surgical planning, patient education and during the procedure for kidney, liver, lung and pancreas surgery became available in the recent years (Intuitive’s Iris, Fujifilm’s SYNAPSE 3D).

### 2.2 Applications of level 1 AR/”What do I see?”

The endoscopic video image and other situational data is analysed and results are displayed in real-time, either superimposed onto the real-world image or next to it.

#### 2.2.1 Instrument/procedure phase detection

An instrument in the image is detected in real-time and related information are displayed (e.g., recommended settings for this instrument).

AI deep learning segmentation models that generate pixel level results are currently investigated for detecting instruments during laparoscopic surgery and technical feasibility has been shown by academic groups (14).

Though applications like displaying tool-specific advice to the user might provide some value to the user, it is most likely the combination with further, complementary data that leads to more impactful solutions, such as surgery phase detection (based on the instrument used) for e.g., automated reporting, performance monitoring and further operational optimization. So far, no such products are available on the market.

Furthermore, tracking of instrument movement throughout particular laparoscopic procedure steps (such as suturing) can be a valuable quality indicator as less movement correlates with higher precision of surgical steps and less traumatic stress for the patient (15).

#### 2.2.2 Anatomical structure detection

Anatomical structures are detected and annotated on the real-world image in real-time. Further value can be generated by combining the anatomical information with e.g., instrument detection results to identify the procedure phase and enable related applications.

AI deep learning detection models that can result in bounding boxes, positioned at the detected structures need to be trained with a sufficient amount of high quality training data for reliable results. For extending the detected structures, the respective AI models need to be re-trained with appropriate training data. Extending the initial model by further, specialized AI models using transfer learning increase the performance of the resulting model (16).

Solutions for detecting anatomical structures have become available in the market in the GI endoscopy space in the recent years (Colon polyp detection: Fuji’s CAD EYE™, Medtronic’s GI Genius™, Olympus’ OIP-1™ and several others. Polyp characterization: Fuji’s CAD EYE^TM,^ NEC’s “WISE VISION^®^ Endoscopy and several others). In laparoscopy, anatomical structures are often hidden behind fatty and fibrous tissue and thereby difficult to detect. A recent study has shown the technical feasibility of anatomical structure detection for key abdominal structures (e.g., critical intestinal vessels to determine safe/no-fly zones), but no commercial products with proven clinical impact are on the market, yet (17, 18).

### 2.3 Applications of level 2 AR/”Where am I?” “Where do I have to go?”

Several sources of information are correlated to support the practitioner in determining the current position in the context of its surroundings (see example in **Table 2**).

#### 2.3.1 Scope position and orientation

The real-time orientation of a flexible endoscope is displayed on the monitor, helping the user to estimate its position in the organ and detect problematic scope deflections early (such as looping).

Magnetic pulses that are sent by the flexible endoscope, and analyzed by an external receiver are enabling the visualization of the current orientation of the scope inside the patient’s body. Several products for this application are available on the market (Fujifilm 3D Scope Imaging System/ColoAssist Pro; Olympus ScopeGuide). While information on the scope orientation is not relevant for rigid scopes, similar technology is employed for tracking scope movement in laparoscopy, mainly for training and skill assessment purposes (Reiley et al.).

#### 2.3.2 Safe/no-fly zone marking

Areas harboring critical structures that are often invisible in an endoscopic image (e.g., bile ducts, nerves or arteries and veins embedded in organ parenchyma) are overlaid onto the field of view in real-time.

Information gathered via other imaging modalities, such as MRI and CT are used to enrich the endoscopic image with information that is only (clearly) visible in the complementary imaging techniques. For generating overlays onto the real-time endoscopic image, a visual representation of the area needs to be generated based on the complementary image information (image reconstruction). This visual representation is then combined with the information of the other modality (image registration and image fusion) to analyze and eventually visualize safe/no-fly tissue patches. High organ deformation in abdominal organs makes accurate real-time registration of pre-procedure imaging data a limiting factor to date, while those systems for rigid organs (neurosurgery, orthopedics) are used in clinical routine today. Developments of soft tissue registration to pre-operative multi-modal imaging data are still in the academic research phase (19, 20). A first step may be leveraging complementary intra-procedure imaging information (such as endoscopic ultrasound (EUS)) for image fusions or overlays to augment the endoscopic image in real-time.

#### 2.3.3 Unobserved area detection

A 3D model of what has been looked at during the procedure is created in real-time and blind spots (i.e., areas that have not been captured by the endoscope) are highlighted or other parameters such as the bowel prep score or the rate of visible mucosa is displayed. For laparoscopic procedures, these applications are less relevant.

Camera pose estimation technologies and AI-enabled Simultaneous Localization and Mapping (SLAM) algorithms are used to generate 3D models from endoscopic images in real-time. An enabling technology for this is real-time monocular depth estimation, currently being in academic research phase (21, 22). Other image analysis algorithms detect and calculate the rate of visible mucosa per endoscopic image or display the estimated bowel prep score (23, 24). While the market for such applications is still very early, there are efforts address the problem of unobserved areas during endoscopy by the means of e.g., mechanically expanding the colon for better visualization by assisted colonoscopy devices that were shown to significantly increase the adenoma detection rate (ADR) (25).

#### 2.3.4 Point of interest localization

Certain structures that are of special relevance for the procedure (e.g., previously detected polyps during colonoscopy) are highlighted to ease (re)discovery. Virtual tags can be placed during surgery to mark areas of interest.

Rediscovery of selected areas can be achieved via topological maps and Bayesian localization. Unlike geometrical maps, topological maps match visual information and do not require a fixed scene geometry. Thereby, they enable matching of imaging data from pliable organs. Current research results on topological maps for endoscopy are encouraging (26), but use in clinical routine requires further development. Virtual tags are under development e.g., by digital surgery companies (27), but not found on the market, yet.

#### 2.3.5 Dynamic 3D organ model

A 3D organ model is displayed on the monitor. The organ model is orienting itself automatically, matching the perspective of the endoscopic image. Instrument 3D models may added and positioned within the organ model.

For generating a 3D organ model, pre-procedure imaging data is reconstructed. Automatic model orientation synced with the endoscopic image requires registration of the model to the real-time endoscopic image. Organ deformation in soft tissues highly complicates successful real-time registration and is the reason for this application being in use for rigid organs, but not for soft tissues in clinical practice to date. Developments of intra-operative soft tissue image registration to pre-operative multi-modal imaging data are still in the academic research phase (20, 28).

## 3 Conclusion and outlook

There are various means to support a practitioner navigate and thereby improve diagnosis and treatment – especially for less experienced practitioners (29, 30). Complexity rises with the analysis and correlation support that helps the practitioner throughout the cognitive navigation loop to answer the questions “What do I know” (e.g., about the patient), “What do I see” (e.g., is this the anatomical landmark I am looking for) up to “Where am I and where do I have to go” (e.g., where is the safe zone/route I can take towards the point of interest).

Foundational technologies that can act as enablers across indications are detection algorithms and transfer learning strategies, SLAM and depth estimation algorithms as well as ways to intuitively provide complementary pre-procedure imaging information during a procedure or even fully automate steps within a surgery or endoscopy procedure. While the latter is quite far out for many GI procedures, e.g., a device for automated suturing for endoscopic gastroplasty is in the clinical development phase (31).

### 3.1 Key aspects for driving adoption of AR navigation solutions

For successfully implementing these AR navigation solutions into the clinical routine and bring advancements to the patients, three key aspects need to be taken into account.

Firstly, there must be a fit to proven clinical workflows. Even the smallest change to how things are done is an additional mental task for a practitioner and a potential distraction. Abrupt, big hardware changes (such as the use of VR headsets in the GI practice or the OR) are very difficult to sustainably implement, augmenting information can easily lead to information-overload and user fatigue (32). AR-assisted seamless workflows are more likely adopted when e.g., “soft robots” are integrated into traditional scope designs, enabling haptic feedback or reducing the need for fine-motor adjustments by the clinician and thereby lessen hand fatigue during lengthy procedures (33). Even without breaking changes on the hardware-side and with intuitive solutions that do not distract the user, physicians need to be trained continuously during this AR-enabled workflow evolution.

Secondly, tech must aim to complement the physician’s capabilities and their value for the user must be imminent (34). While AR navigation systems have the potential to reduce treatment costs by shortening procedure times and realizing other efficiencies (35), their main value will likely lie in quality of care. The developers’ priority aspiration should be to support the practitioner by e.g., lowering the cognitive burden to correlate complementary information. At the same time, users need to understand the system in use. Next to getting educated on key concepts such as data bias, users need to be aware of data generation, usage and ownership flows for a given solution. This at the one hand may reduce the often observed over-reliance on a support system (36). At the other hand this understanding is crucial to build trust. Considering the legal gray areas regarding liability (when is it the system’s fault, when is the physician liable for a wrong decision taken because of a faulty recommendation by an AR navigation system)?, understanding a system’s capabilities but also limitations is extremely important. Current accountability frameworks for digital decision support in medicine, mainly being adapted from autonomous driving use cases, will be translated into actual court decisions step by step, providing more clarity going forward (37). Proper training on the system in use, as well as continuous education on accountability is needed.

Another key aspect is assuring patients’ safety, not only related to the actual procedure, but also related to the data that is generated and used. If hospitals are uncertain about data ownership risks or patients refrain from AR-assisted procedures due to concerns about their sensitive healthcare data, AR navigation systems will face an uphill battle. Transparently complying with all local data privacy and security regulations and enabling users to educate their patients are crucial steps that a supplier must take for successful implementation of an AR navigation solution (38).

### 3.2 User centric design and development

User centricity is the main guiding principle for developers taking these key aspects into account during the development of AR navigation solutions (39). Agility of software development perfectly allows for iterative product development, at least before entering regulatory approval processes. Tech excitement that can easily guide developer teams, constantly needs to be aligned with what is moving the needle for physicians and patients. Developing companies need to gather feedback and usage data whenever possible to learn about pain points, priorities and workflow details to ensure the new technology is integrating as seamless as possible and the value for physician and patient is crystal clear.

## Author contributions

RM: Writing – original draft. PS: Writing – original draft, Writing – review & editing. ZL: Writing – review & editing. AV: Writing – review & editing.
